# Epidemiology of Osteoporosis in Patients with Chronic Obstructive Pulmonary Disease in Taiwan

**DOI:** 10.1007/s44197-023-00183-4

**Published:** 2024-02-14

**Authors:** Kuang-Ming Liao, Chuan-Wei Shen, Kai-Lin Chiu, Chun-Hui Lu, Chih-Wun Fang, Chung-Yu Chen

**Affiliations:** 1 Department of Internal Medicine, Chi Mei Medical Center, Chiali, Taiwan; 2 School of Pharmacy, Kaohsiung Medical University, No. 100, Shiquan 1St Rd., Sanmin Dist., Kaohsiung, 807378 Taiwan; 3 Department of Pharmacy, Kaohsiung Medical University Hospital, Kaohsiung Medical University, Kaohsiung, Taiwan; 4 Institute of Medical Science and Technology, National Sun Yat-Sen University, Kaohsiung, Taiwan; 5 Division of Pharmacy, Zuoying Armed Forces General Hospital, No. 553, Junxiao Rd., Zuoying Dist., Kaohsiung, 813204 Taiwan; 6 Department of Medical Research, Kaohsiung Medical University Hospital, Kaohsiung, Taiwan; 7 INTI International University, Nilai, Malaysia

**Keywords:** Chronic obstructive pulmonary disease, Fracture, Osteoporosis, Epidemiology

## Abstract

**Background:**

Chronic obstructive pulmonary disease (COPD) is a preventable and treatable chronic condition characterized by progressive, partially reversible airflow obstruction. Osteoporosis represents a significant comorbidity in individuals with COPD. However, the incidence and prevalence of osteoporosis among the COPD population remain unclear in Taiwan. Therefore, our objective is to investigate the incidence and prevalence of osteoporosis in patients with COPD.

**Methods:**

In this cross-sectional study, we enrolled a COPD population retrieved from the Taiwan National Health Insurance Research Database (NHIRD) spanning the years 2003 to 2016. Osteoporosis patients were identified using diagnosis codes. The study included newly diagnosed COPD patients from 2003 to 2016. The case group comprised patients who developed osteoporosis or osteoporotic fractures after their COPD diagnosis. We calculated the prevalence and incidence of osteoporosis in individuals with COPD and conducted trend tests.

**Results:**

A total of 1,297,579 COPD patients were identified during the period from 2003 to 2016, with 275,233 of them in the osteoporosis group. The average prevalence of osteoporosis among individuals with COPD was 21.21% from 2003 to 2016 in Taiwan. The number of osteoporosis cases increased from 6,727 in 2003 to 24,184 in 2016. The prevalence of osteoporosis among COPD patients increased from 3.62% in 2003 to 18.72% in 2016. The number of osteoporosis cases among individuals with COPD continued to rise over the years, reaching its highest point in 2016 with 24,184 new cases. The incidence of osteoporosis fluctuated during the study period but generally remained around 3,000 cases per 100,000 person-years. Notably, there was a significant upward trend in incidence from 2003 to 2006, after which the trend stabilized and remained relatively constant.

**Conclusions:**

Our study highlights an increase in both the prevalence and incidence of osteoporosis in individuals with COPD. Given the significant medical, economic, and social implications associated with osteoporosis, a comprehensive and robust assessment of its healthcare burden can offer valuable insights for healthcare system planning and policymaking.

## Background

Chronic obstructive pulmonary disease (COPD) is characterized by progressive airflow limitation caused by a chronic inflammatory response in the lungs and airways [1]. COPD is a significant global cause of mortality and was declared the third leading cause of death by the World Health Organization in 2020 [2]. It is often associated with comorbidities that increase mortality, including osteoporosis, infections, cardiovascular events, lung cancer, and diabetes [3].

Osteoporosis is recognized as one of the major comorbidities in COPD, but it is often underdiagnosed. COPD patients experience symptoms such as shortness of breath, dyspnea, and wheezing, especially during physical activities [4]. Consequently, COPD patients have fewer opportunities to exercise, leading to bone density loss, while inflammatory mediators in circulation may also contribute to skeletal muscle weakness. Another contributing factor is the use of corticosteroids during COPD exacerbations. Numerous studies have shown a link between corticosteroid use and osteoporosis [5, 6]. Glucocorticoid-induced osteoporosis is a common cause of osteoporosis in COPD patients, but they require oral or parenteral corticosteroids to alleviate symptoms during acute exacerbations [3].

The pathology of osteoporosis in COPD is complex, and multiple risk factors may contribute to the high prevalence of osteoporosis, including older age, smoking, systemic inflammation, vitamin D deficiency, and the use of oral corticosteroids [7]. Additionally, osteoporosis is associated with poor health status and prognosis in COPD. Various risk factors can impact disease prognosis and overall health status and survival [8]. However, the range of osteoporosis prevalence in COPD patients varies widely due to differences in diagnosis methods and definitions. Different studies use bone mineral density and diagnosis codes. Some studies may even include osteoporosis and osteoporotic fractures; if patients have had fractures in the past, the risk of osteoporosis may increase. The prevalence of osteoporosis in COPD ranges from 23 to 50% based on bone mineral density diagnosis [9], while the prevalence of osteoporosis and fractures in COPD ranges from 24 to 80% [9]. There is limited data available on osteoporosis in COPD patients in Taiwan. Therefore, we conducted a nationwide, population-based cross-sectional study in Taiwan from 2003 to 2016 to assess the prevalence and incidence of osteoporosis in COPD.

## Methods

### Data Source

Taiwan established the National Health Insurance (NHI) program in 1995, providing coverage to 99.9% of its 23 million population and 93.03% of healthcare providers. The NHI program includes clinical information, such as medical diagnoses, procedures, and dispensed medications in outpatient, inpatient, and emergency settings. We utilized the full population database from the NHI program, covering medical records from January 1, 2002, to December 31, 2017.

### Study Design and Population

We conducted a nationwide, population-based cross-sectional study to assess the prevalence and incidence of osteoporosis in patients diagnosed with COPD. The study included newly diagnosed COPD patients from 2003 to 2016. The case group comprised patients who developed osteoporosis or osteoporotic fractures after their COPD diagnosis.

### Identification of COPD Cohort

#### Inclusion Criteria

1. Patients aged between 40 and 90 years.
2. Newly diagnosed COPD between January 1, 2003, and December 31, 2016 (International classification of diseases, ninth revision, clinical modification (ICD-9-CM) code: 490, 491, 492, 496; international classification of diseases, Tenth Revision, clinical modification (ICD-10-CM) code: J40, J41, J43, J44).
3. Patients with at least one inpatient diagnosis or more than two consecutive outpatient diagnoses of COPD within one year.
4. Patients treated with COPD medications in outpatient claims (COPD medications: long-acting muscarinic antagonists, long-acting beta-agonist, short-acting muscarinic antagonists, short-acting beta-agonist, or oral methylxanthines).

#### Exclusion Criteria

1. COPD patients who developed osteoporosis before the diagnosis of COPD (ICD-9-CM code: 733; ICD-10-CM code: M80, M81, M82).
2. COPD patients who developed osteoporotic fractures, including vertebral fractures, hip fractures, humeral, and radio-ulnar fractures before the diagnosis of COPD (ICD-9-CM: 805.2–805.9, 820, 812, 813; ICD-10-CM: S21, S22, S32, S72, S79, S42 S49, S52, S59).
3. Patients who died within 28 days after the COPD diagnosis.
4. Patients with missing or incomplete data in the NHI program.

### Index Date

The index date was the date of COPD diagnosis (ICD-9-CM code: 490, 491, 492, 496; ICD-10-CM code: J40, J41, J43, J44).

### Outcome Definition

The outcome was defined as the date of the first osteoporosis or osteoporotic fracture event (ICD-9-CM: 733, 805.2–805.9, 820, 812, 813; ICD-10-CM: M80, M81, M82, S21, S22, S32, S72, S79, S42 S49, S52, S59) after diagnosis of COPD.

### Variables

We analyzed the characteristics of COPD patients, including age, age group (ten years as one level), sex, and urbanization (urban, suburban, and rural). Comorbidities were defined as having more than one inpatient diagnosis or more than two outpatient diagnoses within one year before and after the COPD diagnosis date. The ICD-9-cm diagnosis codes and ICD-10 coding for comorbidities were as follows. Asthma: 493 / J452, J453, J454, J459; lung cancer:162/C33, C340, C341, C342, C343, C348, C349, C7A090, Z5112; dyslipidemia: 272/E75, E77-E78; hypertension: 401–405/I10-I15; diabetes mellitus: 250/E10-E14; obesity: 278.0/E661, E662, E663, E668, E669, E6601; chronic kidney disease: 585/N184, N185, N186, N189; chronic liver disease: 070.22, 070.23, 070.32, 070.33, 070.54, 070.59, 070.6, 070.9, 456.0, 456.1, 456.2, 570, 571, 572.2, 572.3, 572.4, 572.8, 573.3, 573.4, 573.8, 573.9, V42.7/K70, K73, K74; hyperthyroidism: 242/E05; malignancy: 140–239/C00–C97, D00–D49; rheumatoid arthritis: 714 / M05; coronary artery disease: 410–414, 429.2/I20-I25; peripheral vascular disease: 440–448/I70, M30, M31; ischemic stroke/transient ischemic attack: 433–437/G45, G46, I63-I68; hemorrhagic stroke: 430–432/I60-I62; heart failure: 398.91, 402.01, 402.11, 402.91, 404.01, 404.03, 404.11, 404.13, 404.91, 404.93, 425.4, 425.7, 425.8, 425.9, 428/I50; left ventricular hypertrophy: 429.3/I51.7; atrial fibrillation: 427/I48.

### Measurement of Covariates

Baseline characteristics included age, age group, gender, urbanization level, insurance premium, comorbidities, and COPD medications. COPD medications were identified from inpatient or outpatient claims. Patients were categorized as urban, suburban, or rural based on their enrolled location in the dataset.

### Severity of COPD

Due to the lack of lung function test data and laboratory exam results in the NHI dataset, we used the refined assessment tool from the GOLD guideline to assess exacerbation risk and COPD severity [10]. The assessment tool divided patients into high-risk and low-risk groups based on the number of exacerbation events in the past year. Severe exacerbations led to emergency room visits or hospital admissions, while moderate exacerbations did not lead to hospital admission but required systemic corticosteroid and/or antibiotic treatment. Patients with ≥ 2 moderate exacerbations or ≥ 1 severe exacerbation in the past year were considered high risk for exacerbation. We used anatomical therapeutic chemical codes to define antibiotics and oral corticosteroids.

### Statistical analysis

#### Prevalence

Osteoporosis prevalence was defined as the proportion of osteoporosis and osteoporotic fracture patients (spine fracture, hip fracture, and humero-radio-ulnar fracture) divided by the number of COPD patients with the same diagnosis year in each year. Prevalence of osteoporosis in the COPD population = (Number of osteoporosis patients) / (Number of COPD population).

#### Incidence

Osteoporosis incidence was defined as the number of newly diagnosed osteoporosis and osteoporotic fracture patients divided by the number of COPD patients at risk in each year, presented as the number per 100,000. For trend analysis, we used the Cochran–Armitage test for trend to analyze if there was a significant change in incidence during the study period. Incidence (per 100,000 person-years) = (Number of newly diagnosed osteoporosis and osteoporotic fracture patients) / (Number of COPD population) × 100,000.

### Statistical Software

Data processing and statistical analysis were performed using SAS 9.4 software. Statistical significance was determined with a two-tailed significance level of α = 0.05.

## Results

### Study Population

**Fig. 1:**
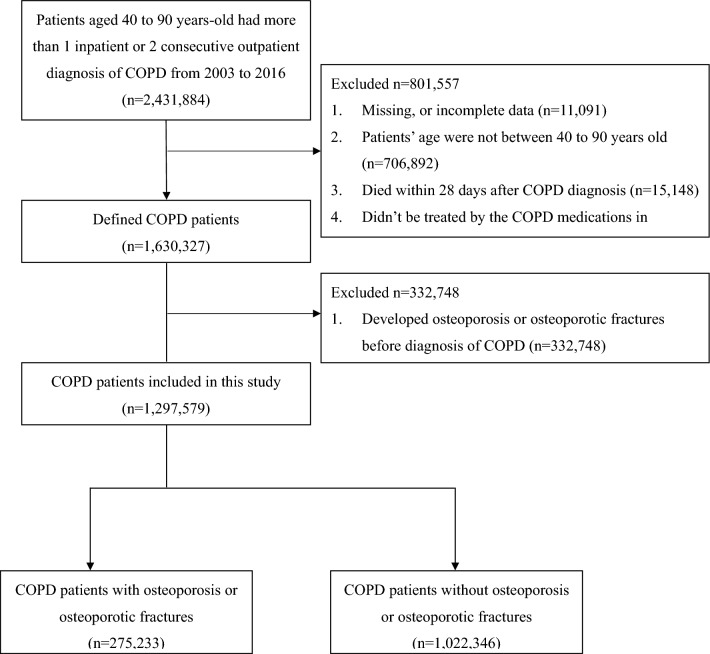
The flowchart of the study population

A total of 2,431,884 COPD patients aged between 40 and 90 years were identified in the database from 2003 to 2016. After confirming COPD diagnosis using admission claims and COPD treatment medication, 1,630,327 COPD patients remained eligible for the study. Based on the inclusion and exclusion criteria, 1,297,579 patients were included in the COPD cohort. Of these, 332,748 patients developed osteoporosis or osteoporotic fractures before the diagnosis of COPD. Figure 1 presents a detailed flowchart of the study population.

The COPD cohort included 275,233 patients with osteoporosis and 1,022,346 patients without osteoporosis. The mean age of patients in the osteoporosis group was 65.62 years old, while in the non-osteoporosis group, it was 60.95 years old. The mean age in the osteoporosis group was higher than in the non-osteoporosis group. Most patients in both groups were between 50 and 70 years old. The distribution of gender in both groups was different, with the majority being female in the osteoporosis group, while the majority was male in the non-osteoporosis group. In terms of medication for COPD treatment, approximately half of the patients in both groups were using systemic beta-2-adrenoreceptor agonists, corticosteroids, and methylxanthines. There was a significant difference in the severity of COPD in moderate and severe exacerbations between both groups (p < 0.001). The majority of patients in both groups had a low exacerbation risk in the year following the COPD diagnosis date. Table 1 provides baseline characteristics one year after cohort entry, including age, age group, gender, urbanization level, insurance premium, and COPD medications.

**Table 1:** Baseline Characteristic of COPD patients in one year after cohort entry

Baseline characteristics N (%)	Osteoporosis (N = 275,233)	Non-osteoporosis (N = 1,022,346)	*p* value
Age mean (SD)	65.62 (11.89)	60.95 (12.74)	< 0.0001
Age group			< 0.0001
40 ≤ age < 50	30,677 (11.15)	227,901 (22.29)	
50 ≤ age < 60	57,047 (20.73)	276,796 (27.07)	
60 ≤ age < 70	72,880 (26.48)	231,839 (22.68)	
70 ≤ age < 80	79,532 (28.90)	189,305 (18.52)	
80 ≤ age	35,097 (12.75)	96,505 (9.44)	
Sex			< 0.0001
Male	128,466 (46.68)	663,368 (64.89)	
Female	146,767 (53.32)	358,978 (35.11)	
Urbanization level			< 0.0001
Urban	134,884 (49.01)	534,889 (52.32)	
Suburban	109,426 (39.76)	385,973 (37.75)	
Rural	30,923 (11.24)	101,484 (9.93)	
No. of COPD exacerbations in one year			
Moderate exacerbations			
0	214,602 (77.97)	767,412 (75.06)	< 0.0001
1	27,545 (10.01)	106,549 (10.42)	
≥ 2	33,086 (12.02)	148,385 (14.51)	
Severe exacerbations			
0	248,353 (90.23)	885,599 (86.62)	< 0.0001
1	19,711 (7.16)	85,949 (8.41)	
≥ 2	7,169 (2.60)	50,798 (4.97)	
COPD exacerbations risk			< 0.0001
Low	226,428 (82.27)	800,243 (78.28)	
High	48,805 (17.73)	222,103 (21.72)	
Medication for COPD			
LABA	7889 (2.87)	30,256 (2.96)	0.0102
LABA/ICS	17,840 (6.48)	74,858 (7.32)	< 0.0001
LAMA	4620 (1.68)	28,124 (2.75)	< 0.0001
LABA/LAMA	170 (0.06)	3248 (0.32)	< 0.0001
SABA	41,970 (15.25)	177,053 (17.32)	< 0.0001
SAMA	27,462 (9.98)	115,887 (11.34)	< 0.0001
SABA/SAMA	14,617 (5.31)	80,277 (7.85)	< 0.0001
Systemic beta-2-adrenoreceptor agonists	144,058 (52.34)	509,052 (49.79)	< 0.0001
ICS	9987 (3.63)	34,289 (3.35)	< 0.0001
Corticosteroid	115,055 (41.80)	409,780 (40.08)	< 0.0001
LABA/LAMA/ICS	0 (0.00)	0 (0.00)	NA
Methyl-xanthines	178,123 (64.72)	621,848 (60.83)	< 0.0001
Antibiotic	165,303 (60.06)	593,828 (58.08)	< 0.0001
Co-medication			
SSRI	1400 (1.66)	6436 (0.69)	< 0.0001
Antiepileptic drug	16,757 (19.86)	79,508 (8.52)	< 0.0001
Chemotherapy	208 (0.25)	1177 (0.13)	< 0.0001
Gonadotropin-releasing hormone agents	15 (0.02)	103 (0.01)	0.0813
Aromatase inhibitors	11 (0.01)	74 (0.01)	0.1197
Lithium	74 (0.09)	271 (0.03)	< 0.0001
Proton pump inhibitors	3639 (4.31)	18,383 (1.97)	< 0.0001
Thiazolidinediones	1186 (1.41)	1896 (0.52)	< 0.0001
Thyroid hormone	423 (0.50)	2,519 (0.27)	< 0.0001
Heparin	132 (0.16)	765 (0.08)	< 0.0001
Immunosuppressants	29 (0.03)	101 (0.01)	< 0.0001
ADT	15 (0.02)	103 (0.01)	0.0813
Aluminum	24,130 (28.59)	115,847 (12.41)	< 0.0001
LMWH	40 (0.05)	228 (0.02)	< 0.0001
Warfarin	671 (0.80)	3685 (0.39)	< 0.0001

*LABA* Long-acting beta-agonist, *ICS* Inhaled corticosteroids, *LAMA* Long-acting muscarinic antagonists, *SABA* Short-acting beta-agonist, *SAMA* Short-acting muscarinic antagonists, *SSRI* Selective serotonin reuptake inhibitors, *ADT* Androgen deprivation therapy, Immunosuppressants cyclosporine or tacrolimus; *LMWH* low-molecular-weight heparin

The osteoporosis group had a significantly higher proportion of patients with rheumatoid arthritis (p < 0.001). Some comorbidities had a significantly higher incidence in the osteoporosis group than in the non-osteoporosis group, such as asthma and hypertension. Dyslipidemia, hyperthyroidism, and hypertension showed similar incidences between the two groups (p = 0.29, p = 0.25, and p = 0.76, respectively). However, within one year after the COPD diagnosis date, hypertension had a significantly higher incidence in the case group. Diabetes mellitus, chronic kidney disease, chronic liver disease, and malignancy had significantly higher proportions in the control group. Table 2 presents the details of comorbidities among COPD patients.

**Table 2:** Baseline comorbidities of COPD patients between osteoporosis and non-osteoporosis

Baseline characteristics N (%)	Osteoporosis (N = 275,233)	Non-osteoporosis (N = 1,022,346)	*p* value
Asthma	6949 (2.52)	23,294 (2.28)	< 0.0001
Dyslipidemia	4210 (1.53)	15,921 (1.56)	0.2968
Hypertension	33,713 (12.25)	124,400 (12.17)	0.25
Diabetes mellitus	18,562 (6.74)	75,099 (7.35)	< 0.0001
Obesity	92 (0.03)	341 (0.03)	0.9854
Chronic kidney disease	3187 (1.16)	14,332 (1.40)	< 0.0001
Chronic liver disease	7254 (2.64)	28,827 (2.82)	< 0.0001
Hyperthyroidism	350 (0.13)	1324 (0.13)	0.7613
Malignancy	8508 (3.09)	46,469 (4.55)	< 0.0001
Rheumatoid arthritis	463 (0.17)	928 (0.09)	< 0.0001
Atherosclerotic cardiovascular disease			
Coronary artery disease	13,615 (4.95)	47,540 (4.65)	< 0.0001
Peripheral vascular disease	1183 (0.43)	5380 (0.53)	< 0.0001
Ischemic stroke/transient ischemic attack	8199 (2.98)	37,554 (3.67)	< 0.0001
Hemorrhagic stroke	1430 (0.52)	11,364 (1.11)	< 0.0001
Heart failure	7746 (2.81)	32,365 (3.17)	< 0.0001
Left ventricular hypertrophy	589 (0.21)	1829 (0.18)	0.0002
Atrial fibrillation	6762 (2.46)	26,191 (2.56)	0.0019

### Prevalence and Incidence

**Fig. 2:**
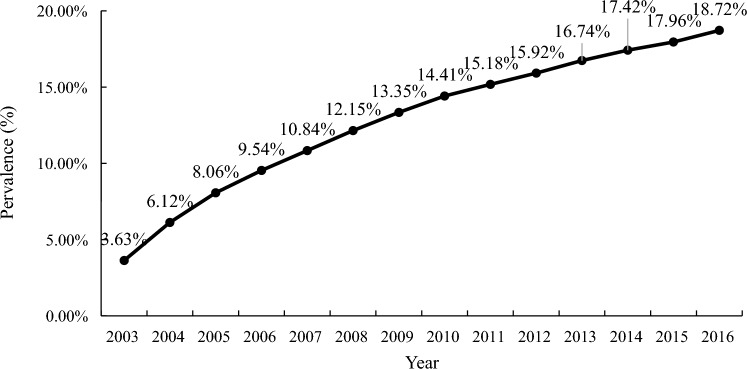
Prevalence of osteoporosis in COPD population during 2003–2016

**Fig. 3:**
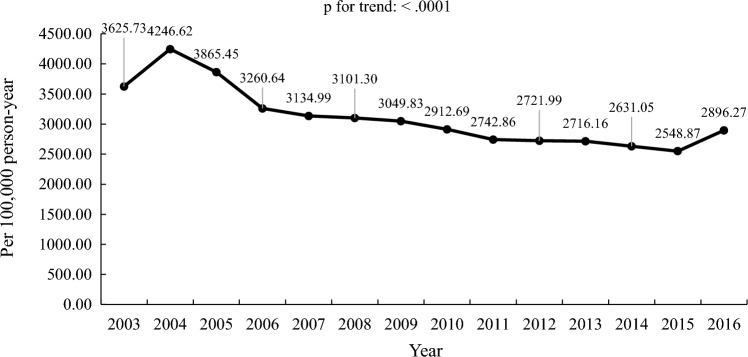
Incidence of osteoporosis in COPD population during 2003–2016

As shown in Table 3, the average prevalence of osteoporosis in the COPD population was 21.21% during 2003–2016. The number of osteoporosis cases increased from 6,727 in 2003 to 24,184 in 2016. The prevalence of osteoporosis increased from 3.62% in 2003 to 18.72% in 2016 (Fig. 2). The annual incidence of osteoporosis exceeded 3,000 person-years, with newly diagnosed osteoporosis patients increasing each year (Table 4). As illustrated in Fig. 3, there was a significant difference in incidence during 2003–2016 according to the trend test (p < 0.0001). The number of osteoporosis cases among COPD patients increased over the years, reaching its highest point in 2016, with 24,184 new cases. The incidence of osteoporosis fluctuated over the years but generally remained about 3,000 cases per 100,000 person-years throughout the study period. Notably, there was a significant upward trend in incidence from 2003 to 2006, after which the trend stabilized and remained relatively constant.

**Table 3:** Prevalence of osteoporosis in COPD population in Taiwan from 2003 to 2016

Year	Number of COPD population			Number of osteoporosis and COPD population			Prevalence of osteoporosis (%)		
	Total	Male	Female	Total	Male	Female	Prevalence	Osteoporosis	COPD
2003	185,535	112,269	73,266	6727	2675	4052	3.63	6727	185,535
2004	150,213	91,849	58,364	13,797	5717	8080	6.12	20,294	331,391
2005	122,405	74,843	47,562	16,366	7013	9353	8.06	35,692	442,718
2006	96,098	59,168	36,930	15,946	7215	8731	9.54	49,871	522,970
2007	91,438	56,616	34,822	17,209	7917	9292	10.84	64,648	596,372
2008	80,191	49,427	30,764	18,434	8521	9913	12.15	79,670	655,632
2009	77,369	47,191	30,178	19,357	9051	10,306	13.35	94,767	710,102
2010	71,797	43,760	28,037	19,453	9125	10,328	14.41	109,178	757,596
2011	75,946	45,908	30,038	19,320	8965	10,355	15.18	122,645	807,700
2012	73,993	45,342	28,651	20,076	9555	10,521	15.92	135,837	853,309
2013	65,894	41,057	24,837	20,665	9760	10,905	16.74	148,783	888,934
2014	67,178	40,641	26,537	20,650	9733	10,917	17.42	161,199	925,406
2015	67,771	40,940	26,831	20,597	9695	10,902	17.96	172,359	959,847
2016	71,751	42,823	28,928	24,184	12,338	11,846	18.72	186,738	997,560
Overall	1,297,579			252,781			21.21

**Table 4:** Incidence of osteoporosis in COPD population in Taiwan from 2003 to 2016

Year	Number of COPD population			Number of osteoporosis and COPD population			Incidence of osteoporosis (per 100,000 person-years)		
	Total	Male	Female	Total	Male	Female	Incidence	Osteoporosis	Person-years
2003	185,535	112,269	73,266	6727	2675	4052	6527.92	6727	103,049.59
2004	150,213	91,849	58,364	13,797	5717	8080	5608.40	13,797	246,005.93
2005	122,405	74,843	47,562	16,366	7013	9353	4595.39	16,366	356,139.66
2006	96,098	59,168	36,930	15,946	7215	8731	3715.68	15,946	429,153.87
2007	91,438	56,616	34,822	17,209	7917	9292	3511.68	17,209	490,050.94
2008	80,191	49,427	30,764	18,434	8521	9913	3420.67	18,434	538,899.66
2009	77,369	47,191	30,178	19,357	9051	10,306	3336.33	19,357	580,187.62
2010	71,797	43,760	28,037	19,453	9125	10,328	3168.30	19,453	613,988.43
2011	75,946	45,908	30,038	19,320	8965	10,355	2970.42	19,320	650,413.11
2012	73,993	45,342	28,651	20,076	9555	10,521	2938.13	20,076	683,291.18
2013	65,894	41,057	24,837	20,665	9760	10,905	2915.92	20,665	708,696.04
2014	67,178	40,641	26,537	20,650	9733	10,917	2820.36	20,650	732,176.99
2015	67,771	40,940	26,831	20,597	9695	10,902	2733.09	20,597	753,614.56
2016	71,751	42,823	28,928	24,184	12,338	11,846	3109.44	24,184	777,760.54
Overall	1,297,579			252,781			3266.68

## Discussion

The study evaluated the epidemiology of osteoporosis in a COPD cohort in Taiwan. In this study, the average prevalence of osteoporosis in COPD was 21.21% from 2003 to 2016. The incidence of osteoporosis in COPD was 3,267 per 100,000 person-years during 2003–2017.

### Prevalence and Incidence of Osteoporosis in COPD

From the NHIRD-related study among the Taiwanese population, the prevalence of osteoporosis was 17.4% in 2001. The incidence rate (per 100,000 person-years) of hip fracture was 277–281 during 2001–2005 and decreased from 262 to 247 in 2006–2011 in the general population [11]. In our study, the prevalence and incidence of osteoporosis in the COPD population were higher than in the general population. Overall, osteoporosis occurred more frequently among COPD patients than in the general population due to various risk factors, such as old age, smoking, lower body mass index, sedentary life style and steroid use [12, 13].

The number of newly diagnosed osteoporosis cases increased each year, from 6,727 in 2003 to 24,184 in 2016. The number was higher in female patients (53.32%) than in male patients (46.68%) in patients with COPD and also observed in general population [14].

The annual incidence of osteoporosis during 2003–2016 was almost consistently above 3,000 person-years. There was an increasing trend in the incidence of osteoporosis during 2003–2016 in Taiwan. Nevertheless, the incidence rate peaked in 2004, after which the incidence trend plateaued and remained around 3,000 per 100,000 COPD person-years. The increase in osteoporosis awareness and policy interventions began in 2003, leading to more people undergoing bone mineral density detection. Consequently, the incidence of osteoporosis peaked in 2004 and then plateaued.

Several general reasons may explain the increased incidence and prevalence in Taiwan's COPD population during the followed period. One of the primary factors is the aging population. Taiwan, like many developed countries, has seen a shift towards an older demographic [15]. As people age, their risk of osteoporosis and related fractures increases. Healthcare systems often respond by prioritizing osteoporosis prevention and management. Additionally, global organizations like the World Health Organization have provided guidelines for osteoporosis prevention and treatment [16], influencing healthcare providers to incorporate therapeutic guidelines and research findings into clinical practice [17]. Besides, improved healthcare access, including bone density testing and early diagnosis, contributes to higher awareness and more diagnoses of osteoporosis [18]. Furthermore, Taiwan's government officially designated osteoporosis as a national health priority, leading to widespread initiatives for prevention and awareness. These efforts have been substantiated by research conducted as part of the National Nutrition and Health Survey in Taiwan [19].

Previous studies have reported a wide range of osteoporosis incidence among COPD patients, from 23% [20] to 69% [21]. In our study, although the incidence of osteoporosis was lower, it is not directly comparable due to differences in data sources. Our study utilized the NHIRD, while related studies were based on hospital-based datasets. Furthermore, our study had a 14-year follow-up period and a sample size of nearly 1.3 million, which differs from previous studies with smaller sample sizes [22–27]. Additionally, most previous studies used dual-energy X-ray absorptiometry scans and T-scores to detect osteoporosis, while our study relied on clinical diagnosis.

The secular changes in osteoporosis in the COPD population in Taiwan are similar to trends observed in Western and Asian countries [28–31]. Our study demonstrated that the prevalence and incidence of osteoporosis in COPD patients were higher than in the general population, highlighting the importance of addressing osteoporosis in the COPD population in Taiwan.

### Strengths and Limitations

To our knowledge, this study is the first to use a nationwide database to explore the epidemiology of osteoporosis in patients with COPD. There are several strengths in our study.

The first strength is the use of the NHIRD, which covers 99.9% of Taiwan's population and 93.03% of healthcare providers. Therefore, our study has a large sample size and is nationally representative, providing sufficient statistical power. The second strength is the long observation time. The study utilized data from 2002 to 2017, providing a 15-year period of follow-up. This extended observation time is crucial for understanding the epidemiology of osteoporosis, as it is a chronic disease with a long development period. Our study reflects real-world clinical data.

However, the study also has limitations. The study population and outcomes are defined based on ICD-9 CM and ICD-10 CM codes due to the lack of examination results and laboratory data in the NHI database. Despite this limitation, we used various alternative definitions that combined diagnosis codes with medication data to improve accuracy. Other limitations of the NHI database include the absence of information on smoking status, lifestyle, lung function, and bone mineral density.

## Conclusion

Our study reveals an elevation in both the prevalence and incidence of osteoporosis among COPD patients. Considering the substantial medical, economic, and social implications of osteoporosis, obtaining a robust and comprehensive assessment of its medical burden can greatly inform healthcare system planning and policymaking. This assessment includes an evaluation of the current situation and future projections, facilitating the provision of sufficient resources for the treatment of patients with COPD and osteoporosis while also addressing the elevated risk of life-threatening outcomes linked to fractures.

## Data Availability

Data are available from the National Health Insurance Research Database (NHIRD) published by Taiwan National Health Insurance (NHI) Bureau. Due to legal restrictions imposed by the government of Taiwan in relation to the “Personal Information Protection Act”, data cannot be made publicly available. Requests for data can be sent as a formal proposal to the NHIRD (http://nhird.nhri.org.tw).

## Abbreviations

COPD: Chronic obstructive pulmonary disease
ICD-9-CM: International classification of diseases, ninth revision, clinical modification
ICD-10-CM: International classification of diseases, Tenth Revision, clinical modification
NHI: National Health Insurance
