# Correction to: Epidemiology of Osteoporosis in Patients with Chronic Obstructive Pulmonary Disease in Taiwan

**DOI:** 10.1007/s44197-024-00248-y

**Published:** 2024-06-03

**Authors:** Kuang-Ming Liao, Chuan-Wei Shen, Kai-Lin Chiu, Chun-Hui Lu, Chih-Wun Fang, Chung-Yu Chen

**Affiliations:** 1 Department of Internal Medicine, Chi Mei Medical Center, Chiali, Taiwan; 2 School of Pharmacy, Kaohsiung Medical University, No. 100, Shiquan 1St Rd., Sanmin Dist, Kaohsiung, 807378 Taiwan; 3 Department of Pharmacy, Kaohsiung Medical University Hospital, Kaohsiung Medical University, Kaohsiung, Taiwan; 4 Institute of Medical Science and Technology, National Sun Yat-Sen University, Kaohsiung, Taiwan; 5 Division of Pharmacy, Zuoying Armed Forces General Hospital, No. 553, Junxiao Rd., Zuoying Dist, Kaohsiung, 813204 Taiwan; 6 Department of Medical Research, Kaohsiung Medical University Hospital, Kaohsiung, Taiwan; 7 INTI International University, Nilai, Malaysia

**Correction to: Journal of Epidemiology and Global Health (2024) 14:213-222**

10.1007/s44197-023-00183-4

When this article was published, the name of the 1st author was regrettably given with a typo. Instead of “Kung-Ming Liao” his correct name is “Kuang-Ming Liao”.

The Original Article has been corrected.

